# Spin-Qubit Noise Spectroscopy of Magnetic Berezinskii–Kosterlitz–Thouless Physics

**DOI:** 10.1021/acs.nanolett.5c04627

**Published:** 2025-12-12

**Authors:** Mark Potts, Shu Zhang

**Affiliations:** † 28269 Max Planck Institute for the Physics of Complex Systems, Nöthnitzer Str. 38, Dresden 01187, Germany; ‡ Collective Dynamics and Quantum Transport Unit, Okinawa Institute of Science and Technology Graduate University, 1919-1 Tancha, Onna-son 904-0495, Japan

**Keywords:** Nitrogen-vacancy magnetometry, Quantum noise spectroscopy, Topological phase transition, Berezinskii−Kosterlitz−Thouless transition, van der Waals magnet, Vortex conductivity

## Abstract

We propose using spin-qubit noise magnetometry to probe dynamical signatures of magnetic Berezinskii–Kosterlitz–Thouless (BKT) physics. For a nitrogen-vacancy (NV) center coupled to two-dimensional XY magnets, we predict distinctive features in the magnetic noise spectral density in the sub-MHz to GHz frequency range. In the quasi-long-range ordered phase, the spectrum exhibits a temperature-dependent power law characteristic of algebraic spin correlations. Above the transition, the noise reflects the proliferation of free vortices and enables quantitative extraction of the vortex conductivity, a key parameter of vortex transport. These results highlight NV as a powerful spectroscopic method to resolve magnetic dynamics in the mesoscopic and low-frequency regimes and to probe exotic magnetic phase transitions.

## Graphical abstract

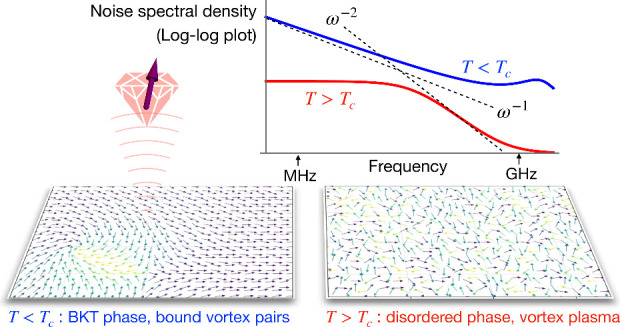

Topology plays a central role in modern condensed matter physics, particularly in identifying exotic phase transitions and unconventional forms of order. A hallmark example is the topological phase transition in two-dimensional XY systems formulated in the seminal works of Berezinskii, Kosterlitz, and Thouless.^1–4^ A transition from quasi-long-range order to disorder is driven by the unbinding of pairs of topological defects, instead of a conventional Landau paradigm transition associated with the breaking of a continuous symmetry, which is forbidden in two dimensions at finite temperature by the Mermin-Wagner theorem.^5^ This BKT transition has since been observed in various physical systems, including thin superconducting^6–8^ and superfluid^9,10^ films, planar arrays of superconductor junctions,^11^ and two-dimensional Bose gases.^12–14^ However, the experimental study of magnetic BKT physics^15–35^ has been hindered by the lack of ideal candidate materials and suitable experimental methods.

Conventional long-range magnetic order tends to form in layered materials, even with weak interlayer coupling, and is also precipitated by magnetic anisotropies in the XY plane. Recent advances in the fabrication of two-dimensional van der Waals magnetic materials^36–38^ have provided promising candidates, including CrCl_3_ ^39–42^ and NiPS_3_,^43–46^ that can be produced as monolayers. These materials possess a hexagonal magnetic planar anisotropy, which is irrelevant in the Kosterlitz–Thouless phase,^47^ suggesting that the magnetic BKT transition may survive.

Noise magnetometry utilizing single-spin qubits, such as nitrogen-vacancy (NV) centers in a diamond, stands out as robust quantum sensor of local magnetic fields, suited to probe dynamics and transport in condensed matter systems.^48–51^ The coupling of magnetic field noise to the NV centers drives both relaxation and dephasing processes, and measurement of the rates of these dynamics allows the extraction of frequency spectra of local magnetic noise. Operating over nm to μm length scales and covering a broad frequency window from kHz to GHz, this approach offers complementary access to magnetic dynamics at meso- and nanoscales—bridging the gap between neutron scattering, optical probes, and transport techniques. It has been proposed and applied to study mesoscopic charge and spin transport,^52–61^ dynamics of topological defects,^54,62–69^ and dynamical phenomena in exotic phases and phase transitions.^70–79^

In this work, we propose leveraging the capabilities of NV noise magnetometry to investigate the dynamical features of magnetic BKT physics. We calculate the magnetic noise spectral density in the MHz ∼ GHz regime, which can be measured by an NV center in proximity to an XY magnet. In the BKT phase, we find a characteristic power-law spectrum with a temperature-dependent exponent, a distinctive hallmark of the algebraic spin correlations intrinsic to the quasi-long-range order. We also predict the functional form of the noise spectrum in the high-temperature disordered phase resulting from spin waves overdamped by free vortices. This offers a noninvasive method to extract the vortex conductivity and quantify vortex dynamics, which can be generally applied to other magnetic systems. The temperature variations in the spectrum clearly capture the proliferation and dynamics of vortices that drive the topological phase transition. Our results highlight the direct access of spin-qubit noise magnetometry to magnetic dynamics in the mesoscopic and low-frequency regimes and promote the use of NV centers as a spectroscopic tool in condensed matter and material studies.

## Results

The NV relaxation and decoherence times (*T*_1_ and *T*_2_) measure the magnetic noise, i.e. temporal fluctuations of the magnetic field, at the position of the NV center that is produced by the spin dynamics within the material system under study. Our main objective is thus to compute the noise spectral density S(ω) from the spin correlations functions of an XY magnet. For a two-dimensional system with axial symmetry, we can separate out the contributions from out-of-plane and in-plane spin components: $S\left(\omega \right)=S_{z}\left(\omega \right)+S_{⊥}\left(\omega \right)$ , where for μ = *z*, ⊥,

$$S_{\mu }\left(\omega \right)=\gamma^{2}f\left(\theta_{NV}\right)\int dk\;k^{3}e^{-2kd}C_{\mu }\left(\omega ,k\right)$$ 1

Here, $C_{z}\left(\omega ,k\right)$ is the Fourier transform of $C_{z}\left(t,r\right)\equiv \left\langle S_{z}\left(t,r\right)S_{z}\left(0,0\right)\right\rangle$ , and $C_{⊥}\left(\omega ,k\right)$ that of $C_{⊥}\left(t,r\right)\equiv \left\langle \underset{i=x,y}{\sum }S_{i}\left(t,r\right)S_{i}\left(0,0\right)\right\rangle /2$ . γ is the gyromagnetic ratio of the magnet, and *k* ^3^ *e* ^–2*kd*^ is the form factor associated with the stray field. *d* is the vertical distance from the NV center to the plane of the XY magnet, which defines the length scale and corresponds to a wavevector *k* ∼ 1/*d* of spin fluctuations to which the probe is most sensitive. The geometric factor *f*(θ_*NV*_) depends on the orientation of the NV spin, and whether we measure the stray field noise transverse or longitudinal to the NV axis, these being accessed by *T*_1_ and *T*_2_ measurements, respectively.

The main results of this work are the distinctive features in the magnetic noise spectrum for the quasi-long-range ordered BKT phase and the high-temperature vortex plasma, as summarized in Figure 1. We plot $\gamma_{NV}^{2}S\left(\omega \right)$ , in units of Hz, to facilitate direct comparison with experiments. The overall factor of γ^2^ *f*(θ_*NV*_) in eq 1 will be implicit in the text from now on. Focusing on the low-frequency regime ω ≪ *c*/*d*, where *c* is the spin wave velocity of the XY magnet, we observe a clear change in spectral behavior above and below the BKT transition:

$$S\left(\omega \right)∼\{\begin{array}{ll} \omega^{\eta -1}, & T≲T_{c},\\ 1/\left(1+\Omega^{2}\omega^{2}/\omega_{s}^{4}\right), & T>T_{c}. \end{array}$$ 2

Ω is an emergent plasma frequency, and ω_*s*_ a resonant spin wave frequency that shall be defined shortly. Below the transition temperature *T*_*c*_, the noise spectrum exhibits a power-law behavior across several orders of magnitude in frequency, as shown in Figure 1 (b). This behavior is inherited directly from the algebraic spin correlations associated with the quasi-long-range order and serves as a clear signature of BKT physics. The exponent of this power law, η – 1, is governed by the dimensionless parameter η = *k*_*B*_ *T*/2*πJ*, with *J* the renormalized spin stiffness in the long-wavelength limit. Approaching the critical point *T*_*c*_, lim_*T*→*T*_*c*_^–^_η = η_*c*_ = 1/4, and the low-frequency exponent drifts from – 1 toward – 3/4.

**Figure 1:**
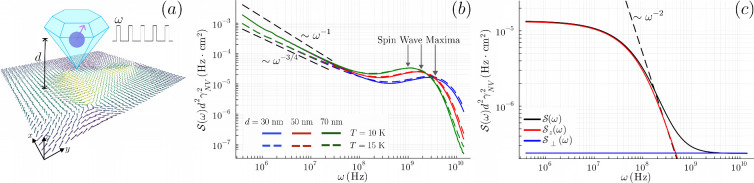
(a) An NV center is placed at a distance *d* above an XY magnet and probes the magnetic noise at frequency ω. (b) The noise spectral density S(ω) below the BKT critical temperature *T*_*c*_ = 15.54 K shows power-law behavior at low frequencies with a temperature-dependent exponent, characteristic of the algebraic spin correlations in the BKT phase. $S\left(\omega \right)d^{2}\gamma_{\mathrm{NV}}^{2}$ collapses for different values of *d*, where γ_NV_ is the gyromagnetic ratio of the NV electron spin. The spin wave maxima are observable at ω ∼ *c*/*d*, where *c* is the renormalized bulk spin-wave velocity. (c) The noise in the disordered phase exhibits a distinctively different frequency dependence. See eq 2. Contributions from the in-plane ( $S_{⊥}$ ) and the out-of-plane spin components ( $S_{z}$ ) alongside the full noise are plotted at *T* = 27.5 K and *d* = 50 nm. We have used realistic material parameters (see main text), solved the renormalization group equations, and performed integrations numerically to produce the results in (b) and (c).

Above *T*_*c*_, spin dynamics are governed by the proliferation of free vortices. Their behavior is analogous to a plasma, with an associated emergent plasma frequency Ω = 2*πσ*/ϵ_*c*_. Here ϵ_*c*_ is the bulk critical value of the *emergent* dielectric constant, and σ = 2*πνJ*_0_ *n*_*f*_ is the vortex “conductivity” [See eq 5] dependent on the free vortex density *n*_*f*_ and the vortex mobility ν. As with propagating light in a traditional plasma, the response of free vortices to spin waves above *T*_*c*_ overdamps these modes at frequencies below Ω, while those at higher frequencies continue to propagate. At temperatures somewhat above *T*_*c*_, where the free vortex density is sufficiently large that the plasma frequency is above the resonance peak ω_*s*_ of the linearly dispersed spin wave, Ω ≫ ω_*s*_ ∼ 5*c*/2*d*, the low-frequency spectral features are generated by overdamped spin wave modes, as given in eq 2 and plotted in Figure 1 (c). Fitting of the measured noise spectrum allows the extraction of the plasma frequency, and hence the vortex conductivity.

## Model

Our analysis of magnetic noise from an XY magnet begins with the following model Hamiltonian:

$$H=\frac{J_{0}}{2S^{2}}\underset{i=x,y}{\sum }\int d^{2}r\;{\left(\nabla S_{i}\right)}^{2}+\frac{1}{2\alpha }\int d^{2}r\;S_{z}^{2}$$ 3

Here *J*_0_ is the exchange stiffness, and α is an easy-plane anisotropy that keeps spins predominantly in the *xy* plane. The *S*_μ_ are coarse-grained spin densities and *S* is the saturated spin (angular momentum) density. For this model to be well described by the planar XY model up to a momentum scale *k*_max_, one requires 1/α ≫ *J*_0_ *k*_max_^2^/*S* ^2^. This can be achieved with only mild magnetic anisotropy (including the single-ion and magnetic dipolar effects) provided one is not probing scales comparable with the lattice spacing *a*_0_.

The azimuthal angle ϕ parametrizes the in-plane spin components *S*_*x*_ = *S*cosϕ and *S*_*y*_ = *S*sinϕ, and its dynamics are accompanied by a small tilt out of the plane *S*_*z*_ = *αϕ̇* (following from the canonical Poisson brackets for spins). This XY magnet can then be mapped to electromagnetism in (2 + 1) dimensions:^3,80–82^ The electric and magnetic fields in terms of the field ϕ­(*t*, **r**)­

$$E=\sqrt{2\pi J_{0}}\nabla \phi \times ẑ,,\mathrm{and},B=\sqrt{2\pi \alpha }\mathrm{\phi ̇}$$ 4

satisfy a complete analog of the Maxwell equations, with charge density ρ and current density **j** proportional to the vortex number density *n*_*f*_ and current **j**_*v*_ respectively.^83^ The equation of motion for ϕ, corresponding to the Ampère-Maxwell law, describes linearly dispersing spin waves, or equivalently an emergent photon, with bare speed $c_{0}=\sqrt{J_{0}/\alpha }$ .

Vortex defects play an essential role in driving the BKT transition, and to model their behavior correctly, ϕ is split into a smooth part θ associated with spin waves, and a singular part ψ associated with the vortices. This is equivalent to a Helmholtz decomposition **E** = **E**_*T*_ + **E**_*L*_, with the transverse component (to the wavevector **k**) being **E**_*T*_ ∝ **∇**θ × **ẑ** and the longitudinal component given by **E**_*L*_ ∝ **∇**ψ × **ẑ**. As vortex cores behave like charges, they have an associated logarithmic interaction energy. Above a critical temperature *T*_*c*_, the gain in entropy for unbinding vortex-antivortex pairs exceeds the unbinding energy cost. The proliferation of free vortices is then responsible for the destruction of the low temperature quasi-long-ranged order.^1–4^

Below *T*_*c*_, the bound vortex pairs serve as instantaneous dipoles, giving the system a finite, scale dependent polarizability, and hence a dielectric constant ϵ­(*r*). This is the central mechanism behind the renormalization group analysis of Berezinskii, Kosterlitz, and Thouless.^1–4,84^ ϵ­(*r*) renormalizes the spin stiffness and the spin wave velocity. The scale dependent dielectric constant can be made dynamical through consideration of the response of vortex pairs to a perturbing potential.^83^ ϵ­(ω, *k*) is calculated by modeling vortex kinetics with the assumption that drag forces originating from the Gilbert damping dominate over the emergent Lorentz force. While in this work we have neglected the spin wave broadening directly due to Gilbert damping, it can in principle be included as a small correction to the imaginary part of ϵ­(ω, *k*). The drifting motion of the (massless) vortices can then be described by a Langevin equation^80^ with an approximately constant vortex mobility ν.^75,85–87^

Above *T*_*c*_, pairs remain bound only within a correlation length $\xi_{+}∼\exp\left(b/\sqrt{T-T_{c}}\right)$ . The dynamical dielectric constant involves a bulk contribution saturated at the critical value ϵ_*c*_ from the remaining bound vortex pairs, and a contribution from the free vortex current:^83^

$$j_{\mathrm{free}}\left(\omega ,k\right)=\sigma \frac{1}{1+iDk^{2}/\omega }E_{L}+\sigma E_{T}$$ 5

Here we define σ = 2*πνJ*_0_ *n*_*f*_ as the vortex conductivity, which is proportional to the density of free vortices *n*_*f*_ ∼ 1/ξ_+_^2^ and, via the Einstein relation, to the vortex diffusion constant *D* = *νk*_*B*_ *T*. The longitudinal electric field is screened by the diffusive vortices, giving an incompressible current in the static limit ω → 0.

## Signatures of the BKT Phase

Following the BKT phenomenology presented above, we apply standard techniques from electromagnetism to obtain the spin-density correlations and the resulting magnetic field noise spectrum.^83^ The starting point is the (retarded) Green’s functions G(ω,k) (isotropic in the *xy* plane) of the vector potential of the fields ([Disp-formula eq4]), which follow directly from Fourier transforming the Maxwell equations. In the low-temperature BKT phase with no free vortices, only the transverse response is present:

$$G_{T}\left(\omega ,k\right)=\frac{2\pi \hbar }{\left[\left(\omega^{2}/c_{0}^{2}\right)\epsilon \left(\omega ,k\right)\right]-k^{2}}$$ 6

Invoking the fluctuation–dissipation theorem in the classical regime *ℏω* ≪ *k*_*B*_ *T* (satisfied for a probing frequency ∼ MHz-GHz and temperature ∼ 10 K), one obtains the correlation function of the transverse vector potential, and hence those of the electromagnetic fields, which can in turn be related to the correlation functions of the order parameter field ϕ and of *S*_*z*_. Using $S_{z}=\sqrt{\alpha /2\pi }B$ , one obtains Cz(ω,k)=(kBTJ0k2/ℏπc02ω)Im⁡GT(ω,k) . As shown in Figure 2.(a), for *T* < *T*_*c*_, $C_{z}\left(\omega ,k\right)$ peaks along the spin wave dispersion with renormalized velocity c(ω)=c0/Reϵ(ω,k→0) (we assume the spin wave wavelength is much larger than the distance $∼\sqrt{D/\omega }$ that vortices can diffuse within the wave period) and broadens as ∼ *k* ^*πJ*/*k*_*B*_ *T*–1^.^80^ This renormalization is relatively weak if a realistic value for the bare vortex chemical potential μ_0_ is taken to be several times of *J*_0_.^3^ Since this intrinsic broadening is much narrower than the width of the wavevector form factor of NV centers, we can approximate $C_{z}\left(\omega ,k\right)$ by a line of delta functions at *k* = ω/*c*(ω), which gives a clean form of the noise spectral density:

$$S_{z}\left(\omega \right)∼\pi k_{B}T\frac{J_{0}}{c_{0}^{2}}\frac{\omega^{3}}{c^{4}\left(\omega \right)}e^{-2\omega d/c\left(\omega \right)}$$ 7

with a maximum at ω ∼ 3*c*(ω)/2*d*, visible in Figure 1 (b).

**Figure 2:**
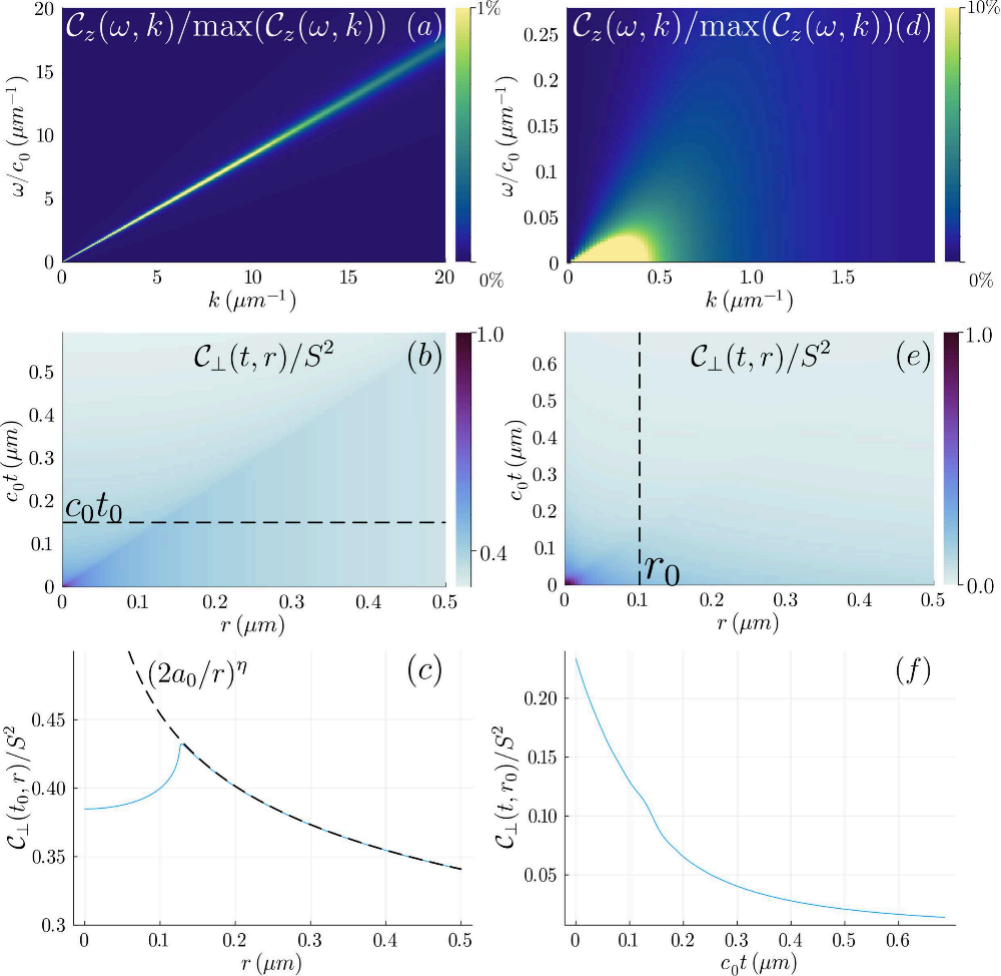
In-plane and out-of-plane spin correlation functions below *T*_*c*_ (a–c) and above *T*_*c*_ (d–f). Here, *J*_0_/*k*_*B*_ ∼ 10 K and μ_0_ ∼ 2*J*_0_ are used to enhance the renormalization effects. (a) Below *T*_*c*_, the out-of-plane spin correlations in the momentum-frequency space $C_{z}\left(\omega ,k\right)$ show a clear linearly dispersed spin wave, which increasingly broadens at higher *k*. (b) Above *T*_*c*_, spin wave modes below the plasma frequency are overdamped by free vortices. Panels (b) and (e) present the in-plane correlations above and below *T*_*c*_. (b) Below *T*_*c*_, the in-plane spin correlations in the real time and space $C_{⊥}\left(t,r\right)$ has a line of maxima alone the c0t/Reϵ(r)=r with power-law decays on either side, as shown in the cut (c). (e) Above *T*_*c*_, the algebraic correlations are washed away, leaving an exponential decay shown in cut (f).

The in-plane spin correlations are computed via $C_{⊥}\left(t,r\right)=S^{2}\exp\left\{-\left\langle {\left[\phi \left(t,r\right)-\phi \left(0,0\right)\right]}^{2}\right\rangle /2\right\}$ . Given that Im ϵ­(ω, *k*) is small,^83^ we have the following analytical expression for the long-wavelength (much larger than lattice spacing *a*_0_) scaling behavior:

$$C_{⊥}\left(t,r\right)\approx S^{2}{\left(\frac{2a_{0}}{r}\right)}^{\eta }\Phi \left(\frac{ct}{r}\right),,\mathrm{where},\Phi \left(u\right)=\{\begin{array}{l} 1,0<u<1,\\ {\left[u+{\left(u^{2}-1\right)}^{1/2}\right]}^{-\eta },u>1. \end{array}$$ 8

Here, $c=c_{0}/\sqrt{\epsilon_{\infty }}$ , where ϵ_*∞*_ is the renormalized bulk dielectric constant. The scaling exponent is given by the dimensionless temperature η = *k*_*B*_ *T*/2*πJ*. $C_{⊥}\left(t,r\right)$ peaks at *r* = *c t* and shows algebraic decay on both sides, characteristic of the quasi-long-ranged order of the low temperature BKT phase [See Figure 2(b, c)]. Numerical integration is performed to obtain the Fourier transform $C_{⊥}\left(\omega ,k\right)$ and the noise spectral density $S_{⊥}\left(\omega \right)$ , which is shown in Figure 1(b). The low-frequency scaling behavior can be seen by an approximation taking *r* ∼ *d* in eq 8, resulting in

$$S_{⊥}\left(\omega \right)∼\frac{\pi S^{2}}{2d^{2}}\int_{0}^{\infty }dt\;\cos\left(\omega t\right){\left(\frac{a_{0}}{ct}\right)}^{\eta }$$ 9

This integral over time yields a power law ω^η–1^. As shown in Figure 1(b), $S_{⊥}\left(\omega \right)d^{2}$ curves at various values of *d* collapse well for ω ≪ *c*/*d*, and show a clean power law across orders of magnitude in frequency, with the temperature dependent exponent η – 1 ∈ [−1, – 3/4]. This spectral dependence serves as a distinct signature for the algebraic spin correlations in the quasi-long-range-ordered BKT phase, in contrast to the magnetic noise spectra below the spin wave gap previously studied in long-range-ordered magnetic systems, where magnon diffusion typically leads to a ω^–2^ dependence,^57,58^ or a peak may arise at a collective magnon hydrodynamic mode from magnon interactions.^59,60^ Such an exponent can also distinguish the BKT phase from the behavior of critical noisy dynamics near continuous phase transitions.^73^ In the clean thermodynamic limit, the zero frequency noise diverges, which is regulated by the finite system size *L*, with the scaling $S_{⊥}\left(\omega \to 0\right)∼L^{1-\eta }$ .^83^

## Vortex Conductivity in the Disordered Phase

We next turn to the disordered phase above *T*_*c*_, where the free vortex-current plays an essential role. The retarded Green’s function of the vector potential now has both transverse and longitudinal components:

$$G_{T}\left(\omega ,k\right)=\frac{2\pi \hbar }{\left[\left(\omega^{2}/c_{0}^{2}\right)\epsilon \left(\omega ,k\right)\right]-k^{2}+i2\pi \sigma \omega /c_{0}^{2}}$$ 10

$$G_{L}\left(\omega ,k\right)=\frac{2\pi \hbar }{\left(\omega^{2}/c_{0}^{2}\right)\left[\epsilon \left(\omega ,k\right)-2\pi \sigma /\left(i\omega -Dk^{2}\right)\right]}$$ 11

For temperatures modestly higher than *T*_*c*_, a constant approximation ϵ­(ω, *k*) ≈ ϵ_*c*_ works well, as the correlation length quickly approaches the scale of *a*_0_ as temperature increases. Below, we use the ϵ_*c*_ in the formulas for brevity, while the frequency and momentum dependence is retained in the numerical computations. We identify a frequency scale in eq 10 that plays the role of a plasma frequency: Ω = 2*πσ*/ϵ_*c*_. For ω < Ω, the dispersive transverse spin wave is predominately relaxational, ω_*T*_ ≈i­(*c*_0_^2^/2*πσ*)*k* ^2^. At long length scales, Ω also determines the relaxation rates of the second transverse mode ω_*T*_ *′* ≈ −iΩ and the longitudinal mode ω = – i­(*Dk* ^2^ + Ω), which is overdamped due to vortex diffusion. Consequently, the spin correlations in the low-frequency regime lose any sharp features with a broadening ∼ Ω.

The out-of-plane spin correlations result only from the spin wave dynamics, Cz(ω,k)=(kBTJ0k2/ℏπc02ω)Im⁡GT(ω,k) , displaying overdamped modes for low frequency [Figure 2 (d)]. Above the plasma frequency ω > Ω, propagating spin waves remain and contribute to the noise spectrum as peaks in the high-frequency, low-temperature regime. On the other hand, vortex diffusion completely removes the algebraic scaling structure from the in-plane spin correlations for length scales above the correlation length^81,87^ (to calculate these for *k* ≲ 1/ξ_+_, the vortex field ψ can be interpreted as arising from a series of vortex multiplets, and is well-defined, allowing one to compute the contribution to in-plane spin correlations from the longitudinal response of the vector potential). The in-plane spin correlations are

$$C_{⊥}\left(t\right)∼S^{2}e^{-4\pi Dn_{f}\ln\left(L/a_{0}\right)t}$$ 12

which decays exponentially in time [Figure 2 (f)] with a lifetime logarithmically diverging with the system size. Indeed, the system is disordered and uncorrelated in any macroscopic scale. In the noise spectrum, the exponential decay gives an extremely broad Lorentzian centered at zero:

$$S_{⊥}\left(\omega \right)∼S^{2}\frac{F\left(a_{0},d\right)}{d^{2}}\frac{W}{\omega^{2}+W^{2}}$$ 13

where the half-width at half-maximum is *W* = 4*πDn*_*f*_ ln­(*L*/*a*_0_) and *F*(*a*_0_, *d*) ≈ (3π/8*d* ^2^)∫_0_^*∞*^d*r r*(1 – 3*r* ^2^/8*d* ^2^)­(1 + *r* ^2^/4*d* ^2^)^−7/2^(2*a*_0_/*r*)^η_*c*_^. This Lorentzian can be seen as a constant plateau for all of the relevant frequencies [Figure 1 (c)], corresponding to the high-frequency plateau in Figure 3, with a temperature-dependent height ∼ 1/*Dn*_*f*_.

**Figure 3:**
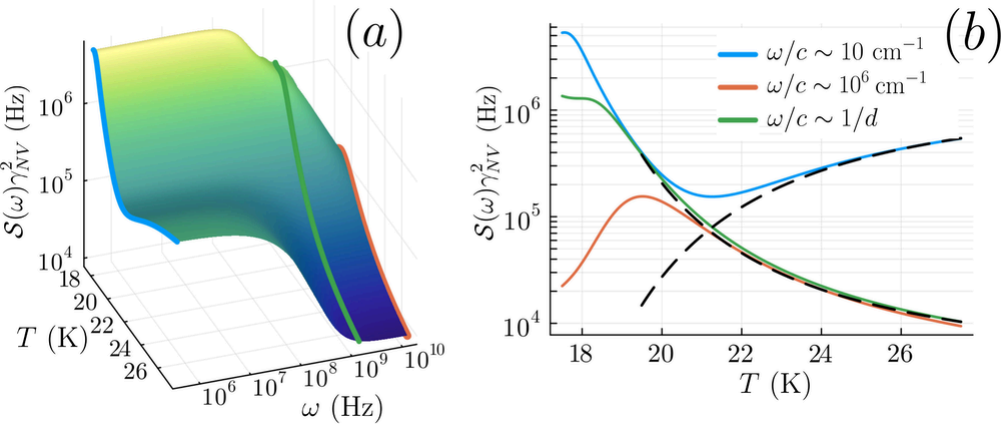
(a) Magnetic noise spectral density for a range of temperatures above *T*_*c*_ and (b) selective cuts at fixed frequencies. *d* = 50 nm is used. Residual spin wave maximum only visible at the low-frequency end of the green curve with ω ∼ *c*/*d* and is absent at higher temperatures. Accompanying the suppression of the spin wave maximum, the spectral weight shifts into a low-frequency plateau with increasing *T*. The dashed lines in (b) show ± log_10_(*Dn*_*f*_) up to a constant shift, which describes the high-temperature behavior of the noise spectrum at low and high frequencies, respectively, inheriting the temperature dependence from the vortex diffusion constant *D* and vortex density *n*_*f*_.

The low-frequency noise spectrum results from the overdamped spin modes in $C_{z}\left(\omega ,k\right)$ . Taking *k* ∼ 5/2*d*, where the momentum form factor *k* ^5^ exp­(−2*kd*) peaks,

$$S_{z}\left(\omega \right)∼\frac{24\pi \sigma k_{B}TJ_{0}}{125d^{2}c_{0}^{4}}\frac{1}{1+\left[{\left(\Omega /\omega_{s}\right)}^{2}-2\right]{\left(\omega /\omega_{s}\right)}^{2}+{\left(\omega /\omega_{s}\right)}^{4}}$$ 14

where $\omega_{s}=5c_{0}/2d\sqrt{\epsilon_{c}}$ . We can therefore fit the measured noise spectrum to this form ([Disp-formula eq14]) with two the fitting parameters Ω and ω_*s*_, besides an overall factor and a constant background, to extract the vortex conductivity σ = (Ω/2π)­(5*c*_0_/2*dω*_*s*_)^2^. In the regime of low-frequency ω ≪ ω_*s*_ and high temperature with a sufficiently large vortex density such that Ω ≫ ω_*s*_, eq 14 reduces to the simpler form presented in the main results eq 2. As shown in Figure 1 (c), the drop in the noise spectrum with increasing frequency is proximate to a ω^–2^ dependence in the window ω_*s*_^2^/Ω ≪ ω ≪ ω_*s*_. The low-frequency plateau, on the other hand approaches the value $S_{z}\left(\omega \to 0\right)$ , which has a temperature dependence following *Dn*_*f*_, as shown in Figure 3. Noting that σ = (2*πJ*_0_/*k*_*B*_ *T*)*Dn*_*f*_, the temperature-dependent plateaus provide an additional reference for the σ extracted from the spectral fitting at different temperatures.

## Predictions for a van der Waals Ferromagnet

To provide quantitative predictions for an experimental NV measurement, Figure 1 and 3 are plotted with material parameters relevant for a van der Waals ferromagnet. We take the bare spin stiffness *J*_0_/*k*_*B*_ ∼ 10 K, and a conservatively small eas*y*-axis anisotropy *ℏ* ^2^/*αa*_0_^2^ *k*_*B*_ ∼ 0.32 K, where the lattice constant *a*_0_ ∼ 6 Å. For example, the anisotropy is estimated to be ∼ 1.3 K for NiPS_3_ ^46^ and ∼ 6.3 K for TmMgGaO_4_.^24^ Here, the bare spin wave velocity is *c*_0_ ∼ 1.4 × 10^4^ cm/s and the BKT transition temperature is *T*_*c*_ = 15.54 K. The bare vortex chemical potential is set at μ_0_ ∼ π^2^ *J*_0_,^3^ and the vortex density is computed by running the renormalization group flow. The vortex mobility is taken to be ν ∼ 6.1 × 10^9^ s/g,^87^ yielding a vortex diffusion constant *D* ∼ 2.3 × 10^–5^ cm^2^/s at *T* = 27.5 K. In Figure 1 (c), we have ω_*s*_ ∼ 7.0 GHz, Ω ∼ 54.7 GHz, and σ ∼ 8.8 GHz. For an experimentally measured noise spectral curve, these parameters can be obtained fitting to eq 14. In the kHz-GHz frequency window, the magnetic noise signal has an order of magnitude of 10^4^–10^7^ Hz, which is well within the experimental reach.

## Discussion

We have demonstrated that spin-qubit magnetic-noise spectroscopy offers a unique probe to identify the dynamical features in magnetic BKT physics. This technique directly accesses the algebraic spin correlations and spin-wave excitations of the quasi-long-ranged order of the BKT phase, and further enables quantitative measurement of the vortex conductivity in the disordered regime. Experimentally, promising platforms are monolayer van der Waals magnets with hexagonal lattice symmetry. Although these systems are expected to undergo a magnetic ordering transition that spontaneously breaks the 6-fold symmetry, the BKT transition may occur at a temperature higher than this,^88^ and the XY-type of dynamics can be relevant for a wider temperature range at finite wavevectors.^34^ Importantly, the prominent wavevector under probe is set by the NV–sample distance, allowing access the low-frequency features while insensitive to lattice-specific details. Our theoretical framework extends naturally to antiferromagnetic systems, and the predicted behaviors for the magnetic noise remain qualitatively valid for systems with an Néel order parameter that does not enlarge the crystallographic unit cell.

A small magnetic field needs to be applied along the NV axis to tune the NV resonance frequency. Due to the sensitivity of the BKT transition is to in-plane magnetic fields that break U(1) symmetry, the preferred measurement geometry is to align the NV axis to the z direction. Noting that the energy scale for the applied fields is of the order of GHz ∼ 0.01K,^89^ much lower than typical magnetic exchange and anisotropy energies of the order THz ∼ 10K,^24,46^ the BKT transition will not be greatly affected.^90–93^

Finally, we contrast the magnetic-noise signatures of BKT physics in magnets with those in two-dimensional superconductors. A recent study^75^ demonstrates that NV magnetometry can probe the dynamical dielectric function across the superconducting BKT transition, where magnetic noise is dominated by electric current fluctuations and exhibits a cusp-like feature. In the magnetic case, however, the noise arises from the dynamics of the order parameter itself, and no sharp singularity is expected at the transition. Instead, the rapid growth of the free-vortex density drives a steep increase in the plasma frequency, leading to a pronounced redistribution of spin-wave spectral weight from high to low frequencies. The spectral dependence enables direct characterization of the scaling behavior of spin correlations in the low-temperature phase and vortex transport phenomena in the high-temperature phase. These features establish NV-based measurements in magnetic systems as a powerful spectroscopic tool to quantitatively resolve dynamical properties and reveal their changes across phase transitions, complementary to the thermodynamic singularities.

## Supplementary Material



## Notes

(I) A review of the formulation of the XY model in terms of emergent electromagnetism in two-dimensions; (II) details of the kinetic theory used to describe the dynamics of vortices in this work; (III) details of the linear response theory used to determine spin correlation functions; and (IV) a short discussion on naive finite size effects on algebraic correlations.^93,94^ (PDF)
