# Supplementary material for: Spin-Qubit Noise Spectroscopy of Magnetic Berezinskii–Kosterlitz–Thouless Physics
Source: Nano Lett. 2025 Dec 12;25(51):17677–84. doi: 10.1021/acs.nanolett.5c04627 (PMC12751109; doi:10.1021/acs.nanolett.5c04627)
Supplement: Supplementary file 1 [file nl5c04627_si_001.pdf]

Spin-qubit Noise Spectroscopy of Magnetic Berezinskii-Kosterlitz-Thouless Physics

Mark Potts[1] and Shu Zhang[2,1]

[1] *Max Planck Institute for the Physics of Complex Systems, Nöthnitzer Str. 38, Dresden 01187, Germany*

[2] *Collective Dynamics and Quantum Transport Unit, Okinawa Institute of Science and Technology Graduate University, 1919-1 Tancha, Onna-son 904-0495, Japan*

I. EMERGENT MAXWELL EQUATIONS

In this section we present a review of how the $(2+1)$d electromagnetic description of the XY model is obtained. We broadly follow the treatment found in Ref. [1].

Starting from our model Hamiltonian:

$$H = \frac{J_0}{2S^2} \sum_{i=x,y} \int \mathrm{d}^2\mathbf{r}\, (\nabla S_i)^2 + \frac{1}{2\alpha} \int \mathrm{d}^2\mathbf{r}\; S_z^2, \tag{S1}$$

one can write down the equations of motion for the azimuthal angle variable ϕ and its conjugate momentum $S_z = \Pi_\phi \equiv \alpha\dot{\phi}$ as:

$$\dot{\Pi}_\phi = J_0 \nabla^2 \phi, \tag{S2}$$

$$\dot{\phi} = \frac{1}{\alpha}\Pi_\phi. \tag{S3}$$

We can rewrite the first of these as a wave equation:

$$\ddot{\phi} = \frac{J_0}{\alpha}\nabla^2\phi, \tag{S4}$$

describing the propagation of spin-wave excitations with bare speed $c_0 = \sqrt{J_0/\alpha}$.

Vortices are topological defects (homotopy defects) in the field ϕ. To include their effects on the dynamics, one separates $\phi(\mathbf{r})$ into a non-compact field $\theta(\mathbf{r})$ associated with spin-waves, and a field $\psi(\mathbf{r})$ associated with the winding of ϕ about vortex cores. The field about a positive vortex centered at the origin is given by:

$$\psi(\mathbf{r}) = \arctan\left(\frac{y}{x}\right). \tag{S5}$$

If one inserts vortices with sign $n_i = \pm 1$ at points $\mathbf{r}_i$, and then take the gradient of the resulting expression, one obtains:

$$\begin{aligned}
\psi(\mathbf{r}) &= \sum_i n_i \arctan\left(\frac{y-y_i}{x-x_i}\right), \\
\nabla\psi &= \sum_i n_i \nabla \arctan\left(\frac{y-y_i}{x-x_i}\right), \\
&= \sum_i n_i \frac{1}{|\mathbf{r}-\mathbf{r}_i|^2} \left\{(x-x_i)\hat{e}_y - (y-y_i)\hat{e}_x\right\}, \\
&= \sum_i n_i \hat{\mathbf{e}}_z \times \nabla G(\mathbf{r}-\mathbf{r}_i).
\end{aligned}$$

In the last line, the expression is rewritten in terms of the Green's function for the Laplacian in two dimensions, which satisfies:

$$\nabla^2 G(\mathbf{r}-\mathbf{r}_i) = 2\pi\delta(\mathbf{r}-\mathbf{r}_i). \tag{S6}$$

This Green's function is given by $\ln(|\mathbf{r}-\mathbf{r}_i|)+c$. If each vortex has a core radius of the order a_0, then $G(r)\sim\ln(r/a_0)$. As the curl of a gradient vanishes, one has that the vortex field satisfies $\nabla\cdot\nabla\psi=0$. The vortex field is purely transverse, and trivially $\nabla\theta$ – the spin-wave part of $\nabla\phi$ – is purely longitudinal, allowing the Helmholtz decomposition:

$$\nabla\phi=\nabla\theta+\nabla\psi. \tag{S7}$$

From stokes' theorem, one can show that the curl of $\nabla\psi$ does not vanish due to the singularity at the center of each vortex, and is hence not a pure gradient. One instead finds that it satisfies:

$$\nabla\times\nabla\psi=\sum_i n_i 2\pi\delta(\mathbf{r}-\mathbf{r}_i)\hat{\mathbf{e}}_z\equiv 2\pi n(\mathbf{r})\hat{\mathbf{e}}_z, \tag{S8}$$

where in the last line the vortex density $n(\mathbf{r})$ is defined.

In taking the time derivative of the vortex field gradient, one must ensure the dynamics of the field singularities are properly accounted for. We define a vortex current $\mathbf{j}_v$ through the continuity equation:

$$\frac{\mathrm{d}n}{\mathrm{d}t}+\nabla\cdot\mathbf{j}_v=0. \tag{S9}$$

Taking the time derivative of Eq. (S8), and then integrating over a curve around a vortex, one finds:

$$\begin{aligned}\frac{\mathrm{d}}{\mathrm{d}t}(\nabla\times\nabla\psi)\cdot\hat{\mathbf{e}}_z&=-2\pi\nabla\cdot\mathbf{j}_v,\\ \int_A\frac{\mathrm{d}}{\mathrm{d}t}(\nabla\times\nabla\psi)\cdot\mathrm{d}\mathbf{A}&=-2\pi\int_A\nabla\cdot\mathbf{j}_v\mathrm{d}A,\\ \oint_{\partial A}\frac{\mathrm{d}}{\mathrm{d}t}\nabla\psi\cdot\mathrm{d}\mathbf{l}&=-2\pi\oint_{\partial A}\mathbf{j}_v\cdot\mathrm{d}\mathbf{s}.\end{aligned}$$

The line elements $\mathrm{d}\mathbf{s}$ and $\mathrm{d}\mathbf{l}$ are related by $\mathrm{d}\mathbf{s}=\hat{\mathbf{e}}_z\times\mathrm{d}\mathbf{l}$. Now equating the integrands of the last line to get the singular part of the time derivative, one finds that the contribution from the vortex cores is $-2\pi(\hat{\mathbf{e}}_z\times\mathbf{j}_v)$. The full time derivative is hence:

$$\frac{\mathrm{d}}{\mathrm{d}t}\nabla\psi=\nabla\left(\frac{\mathrm{d}\psi}{\mathrm{d}t}\right)-2\pi(\hat{\mathbf{e}}_z\times\mathbf{j}_v). \tag{S10}$$

The results so far obtained can be massaged into a form reminiscent of Maxwell's equations:

$$\nabla\cdot(\nabla\phi\times\hat{\mathbf{e}}_z)=2\pi n(\mathbf{r}), \tag{S11}$$

$$\nabla\times(\nabla\phi\times\hat{\mathbf{e}}_z)==-\frac{1}{J_0}\Pi_\phi\hat{\mathbf{e}}_z, \tag{S12}$$

$$\nabla\times(\Pi_\phi\hat{\mathbf{e}}_z)=\alpha\frac{\mathrm{d}}{\mathrm{d}t}(\nabla\phi\times\hat{\mathbf{e}}_z)+2\pi\alpha\mathbf{j}_v. \tag{S13}$$

To make the analogy with electromagnetism clear, one can define an electric field proportional to $\nabla\phi\times\hat{\mathbf{e}}_z$, and a magnetic field proportional to $\Pi_\phi\hat{\mathbf{e}}_z$ and thus recover the Maxwell equations in two dimensions. We define new quantities:

$$\mathbf{E}=\sqrt{2\pi J_0}\nabla\phi\times\hat{\mathbf{e}}_z, \tag{S14}$$

$$\mathbf{B}=\sqrt{\frac{2\pi}{\alpha}}\Pi_\phi\hat{\mathbf{e}}_z, \tag{S15}$$

$$\rho(\mathbf{r})=\sqrt{2\pi J_0}\,n(\mathbf{r}), \tag{S16}$$

$$\mathbf{j}(\mathbf{r})=\sqrt{2\pi J_0}\,\mathbf{j}_v(\mathbf{r}), \tag{S17}$$

and using these definitions, the dynamical equations for spin-waves and vortices are mapped precisely to the Maxwell equations:

$$\nabla \cdot \mathbf{E} = 2\pi\rho(\mathbf{r}), \tag{S18}$$

$$\nabla \cdot \mathbf{B} = 0, \tag{S19}$$

$$\nabla \times \mathbf{E} = -\frac{1}{c_0}\frac{\partial \mathbf{B}}{\partial t}, \tag{S20}$$

$$\nabla \times \mathbf{B} = \frac{1}{c_0}\frac{\partial \mathbf{E}}{\partial t} + \frac{2\pi}{c_0}\mathbf{j}(\mathbf{r}). \tag{S21}$$

II. KINETIC THEORY FOR BOUND AND FREE VORTICES

In this section, we provide more detail on our treatment of the kinetics of free and bound vortices used in our analysis, which primarily follows work presented in [2–4].

One can make use of the electromagnetic analogy to write down the equation of motion for a single vortex. This reads:

$$m\ddot{\mathbf{r}} = 0 = q_i\sqrt{2\pi J_0}\mathbf{E} + \frac{q_i}{c_0}\sqrt{2\pi J_0}\dot{\mathbf{r}} \times \mathbf{B} - C\mathbf{v} - C'\hat{\mathbf{z}} \times \mathbf{v}. \tag{S22}$$

The forces on a vortex must balance as they possess no mass [1]. q_i is the sign of the vortex (clockwise or anticlockwise winding/ positive or negative charge). The ratio of the magnitude of the electric and magnetic forces is the ratio of the spin-wave velocity c_0 to the vortex speed v as expected for charged particles. C and C' parameterize drag forces acting on the vortex. Microscopically, these arise from Glibert spin damping. In the treatment in [4], these drag coefficients are calculated directly from the canonical equations of motion of damped classical spins, where it is shown that $C' \approx 0$, and that $C \approx S\delta \log(L/a_0)$, with S the magnitude of the spin density, δ the Gilbert spin-damping parameter, L a macroscopic length scale, and a_0 the microscopic lattice spacing.

Comparing the contributions of the Lorentz force and the linear drag force, one finds that they contribute in the ratio $\sim |B|/S$. Our underlying assumption in this work is that spin dynamics are predominantly confined to the plane, and in this limit $B \propto S_z$. Hence, the ratio of the Lorentz and drag forces must also be small. Gilbert damping also in principle effects the dynamics of propagating spin waves, inducing a small frequency dependent spin-wave lifetime scaling with $\delta\omega$. Its contribution will not be affected by the unbinding of vortices at the BKT transition, and so provided this damping is sufficiently small that spin-waves are not overdamped below T_c, we can make the simplification of dropping this term without affecting the physics.

With these considerations, one arrives naturally at the following drift velocity by dropping the Lorentz force contribution:

$$\mathbf{v}_d = q_i\frac{\sqrt{2\pi J_0}}{C}\mathbf{E}. \tag{S23}$$

Introducing a stochastic element to the motion via a Gaussian noise term $\eta(t)$, one arrives at the following Langevin equation:

$$\frac{\mathrm{d}\mathbf{r}_v}{\mathrm{d}t} = q_i\frac{\sqrt{2\pi J_0}D}{k_B T}\mathbf{E} + \eta(t), \tag{S24}$$

where $1/C$ has been re-parameterized as a mobility $D/k_BT = \nu$. Equation S24 then serves as the basis for the kinetic theory of vortices used in our calculations, determining the linear response of bound pairs of vortices to changes in the surrounding spin configuration [2, 3, 5], and the equilibrium current density in the high temperature phase [3].

Looking first at bound pairs, let us define the probability distribution $P(\mathbf{r}, \mathbf{R}, t)$ as the probability density for a pair of singly charged vortices of center-of-mass position $\mathbf{R}$ to have separation $\mathbf{r} = \mathbf{r}_p - \mathbf{r}_n$. The relevant

Langevin equations for $\mathbf{r}$ and $\mathbf{R}$ are:

$$\frac{\mathrm{d}\mathbf{r}}{\mathrm{d}t} = \frac{\sqrt{2\pi J_0}D}{k_B T} \left[\mathbf{E}(\mathbf{r}_1) + \mathbf{E}(\mathbf{r}_2)\right] + \eta_r(t), \tag{S25}$$

$$\frac{\mathrm{d}\mathbf{R}}{\mathrm{d}t} = \frac{\sqrt{2\pi J_0}D}{2k_B T} \left[\mathbf{E}(\mathbf{r}_1) - \mathbf{E}(\mathbf{r}_2)\right] + \eta_R(t), \tag{S26}$$

with $\langle \eta_r^\alpha(t)\eta_r^\beta(t')\rangle = 4D\delta^{\alpha\beta}\delta(t-t')$, and $\langle \eta_R^\alpha(t)\eta_R^\beta(t')\rangle = D\delta^{\alpha\beta}\delta(t-t')$. Let us now also introduce a perturbing field $\delta\mathbf{E}(\mathbf{r},t) = i\mathbf{k}\delta V e^{\mathrm{i}\mathbf{k}\cdot\mathbf{r}}e^{-\mathrm{i}\omega t}$ to which we will calculate the linear response of the polarization (and hence the contribution to the dynamical dielectric constant). Including the coulomb interaction between the two vortices as well as this perturbation, the above Langevin equations become:

$$\frac{\mathrm{d}\mathbf{r}}{\mathrm{d}t} = \frac{2\sqrt{2\pi J_0}D}{k_B T} \left[-\frac{\sqrt{2\pi J_0}\mathbf{r}}{r^2\epsilon(r)} + i\mathbf{k}\delta V(\mathbf{R},t)\cos\left(\frac{\mathbf{k}\cdot\mathbf{r}}{2}\right)\right] + \eta_r(t), \tag{S27}$$

$$\frac{\mathrm{d}\mathbf{R}}{\mathrm{d}t} = -\frac{\sqrt{2\pi J_0}D}{k_B T}\mathbf{k}\delta V(\mathbf{R},t)\sin\left(\frac{\mathbf{k}\cdot\mathbf{r}}{2}\right) + \eta_R(t). \tag{S28}$$

Here $\epsilon(r)$ is the scale dependent dielectric function derived from the BKT renormalization-group equations [6–8]. The corresponding Fokker-Planck equation for the probability distribution P is then:

$$\begin{aligned}\frac{\partial P}{\partial t} &= \nabla_\mathbf{r}\cdot\left\{\left(\frac{4\pi J_0 D\mathbf{r}}{k_B T r^2\epsilon(r)} - \frac{2D\sqrt{2\pi J_0}i\mathbf{k}\delta V}{k_B T}\cos\left(\frac{\mathbf{k}\cdot\mathbf{r}}{2}\right)\right)P\right\}\\ &+ \nabla_R\cdot\left\{\left(\frac{\sqrt{2\pi J_0}D}{k_B T}\mathbf{k}\delta V\sin\left(\frac{\mathbf{k}\cdot\mathbf{r}}{2}\right)\right)P\right\}\\ &+ 2D\frac{\partial^2 P}{\partial r^2} + \frac{D}{2}\frac{\partial^2 P}{\partial R^2}.\end{aligned} \tag{S29}$$

We now separate out P into the equilibrium distribution P_0, and the response $\delta P = \delta P(\mathbf{r})e^{\mathrm{i}\mathbf{k}\cdot\mathbf{R}}e^{-\mathrm{i}\omega t}$. Keeping only terms linear in δ, one obtains:

$$\begin{aligned}-\mathrm{i}\omega\delta P =& \nabla_r\cdot\left\{\frac{4\pi J_0 D\mathbf{r}}{k_B T r^2\epsilon(r)}\delta P\right\} - i\frac{2D\sqrt{2\pi J_0}}{k_B T}k\delta V\cos\Theta\frac{\partial}{\partial r}\left\{\cos\left(\frac{\mathbf{k}\cdot\mathbf{r}}{2}\right)P_0\right\}\\ &+ \mathrm{i}k^2\frac{\sqrt{2\pi J_0}D}{k_B T}\delta V\sin\left(\frac{\mathbf{k}\cdot\mathbf{r}}{2}\right)P_0\\ &+ 2D\frac{\partial^2\delta P}{\partial r^2} - \frac{Dk^2}{2}\delta P.\end{aligned} \tag{S30}$$

The angle Θ here is that between the perturbing field (or equivalently $\mathbf{k}$) and the relative position vector $\mathbf{r}$. To further simplify this differential equation, one can make the following substitution for δP:

$$\delta P = 2\mathrm{i}\frac{\sqrt{2\pi J_0}}{k_B T}\sin\left(\frac{\mathbf{k}\cdot\mathbf{r}}{2}\right)\delta V P_0 g(r,\Theta,\omega,k). \tag{S31}$$

Inserting this into Eq. (S30), one can make use of the fact that P_0 satisfies:

$$0 = \frac{4\pi J_0 D}{k_B T}\nabla_\mathbf{r}\cdot\left\{\frac{\mathbf{r}P_0}{r^2\epsilon(r)}\right\} + 2D\frac{\partial^2 P_0}{\partial r^2}, \tag{S32}$$

which implies $\partial_r P_0 = -(P_0/k_B T)\partial_r U = -2\pi J_0 P_0/k_B T r\epsilon(r)$, to simplify the differential equation down to:

$$\begin{aligned}r^2 g'' + rg'&\left\{2\left(\frac{\mathbf{k}\cdot\mathbf{r}}{2}\right)\cot\left(\frac{\mathbf{k}\cdot\mathbf{r}}{2}\right) - \frac{2\pi J_0}{k_B T\epsilon(r)}\right\}\\ &+ (1-g)\left\{\frac{k^2r^2}{4}(1+\cos^2\Theta) + \frac{2\pi J_0}{k_B T\epsilon(r)}\left(\frac{\mathbf{k}\cdot\mathbf{r}}{2}\right)\cot\left(\frac{\mathbf{k}\cdot\mathbf{r}}{2}\right)\right\} + \frac{\mathrm{i}\omega r^2}{2D}g = 0.\end{aligned} \tag{S33}$$

g' and g'' indicate partial differentiation with respect to r. This equation is subject to the boundary conditions $g(0) = 1$ and $\lim_{z\to\infty} g(z) = 0$, and if one seeks only static solutions ($\omega = 0$), then $g = 1$ is the unique solution, which recovers the results of Ref. [5]. If we take the limit $\mathbf{k} \to 0$, in principle we should recover the differential equation obtained in Ref. [2]. However we find instead the following slightly different equation:

$$r^2 g'' + rg'\left\{2 - \frac{2\pi J_0}{k_B T\epsilon(r)}\right\} - g\left\{-\frac{\mathrm{i}\omega r^2}{2D} + \frac{2\pi J_0}{k_B T\epsilon(r)}\right\} + \frac{2\pi J_0}{k_B T\epsilon(r)} = 0. \tag{S34}$$

The only difference between the equation arrived at in Ref. [2] and ours is that the '3' in the term proportional to g' is replaced with a '2' in our equation. The method of analysis is identical here to as presented in Ref. [2], and so we attribute the discrepancy to an error made in the original paper. As in that paper, we can further approximate Eq. (S34) by replacing the combination $2\pi J_0/k_B T\epsilon(r)$ by its bulk critical value of 4, and then defining $z^2 = -\mathrm{i}\omega r^2/2D$, giving:

$$z^2 g'' - 2zg' - (z^2 + 4)g + 4 = 0. \tag{S35}$$

The dynamical dielectric constant $\epsilon(\omega, \mathbf{k})$ is defined in terms of the change in the distribution function δP as [2]:

$$\epsilon(\omega, k) = 1 + 4\pi\sqrt{2\pi J_0}\int \mathrm{d}^2\mathbf{r}\frac{\mathbf{r}\cdot\mathbf{k}}{2\mathrm{i}k^2}\frac{\delta P}{\delta V}. \tag{S36}$$

Inserting Eq. (S31) into the above equation, and using the self-consistency equation for the scale-dependent dielectric constant $\epsilon(r)$ [6–8], one obtains:

$$\epsilon(\omega, k) = 1 + \int_{a_0}^{\infty}\mathrm{d}r\frac{\mathrm{d}\epsilon(r)}{\mathrm{d}r}\int_0^{2\pi}\mathrm{d}\Theta\left[\frac{2\cos\Theta}{\pi kr}\sin\left(\frac{kr\cos\Theta}{2}\right)\right]g(r, \Theta, \omega, k). \tag{S37}$$

In the main text, we make the same approximation as made in Ref. [5], and evaluate g in the $\mathbf{k} \to 0$ limit, wherein it is independent of both k and θ, and the above expression simplifies to:

$$\epsilon(\omega, k) = 1 + \int_{a_0}^{\infty}\mathrm{d}r\frac{\mathrm{d}\epsilon(r)}{\mathrm{d}r}\left[\frac{4}{kr}\mathcal{J}_1\left(\frac{kr}{2}\right)\right]g(r, \omega) \approx 1 + \int_{a_0}^{\infty}\mathrm{d}r\frac{\mathrm{d}\epsilon(r)}{\mathrm{d}r}e^{-k^2r^2/32}g(r, \omega). \tag{S38}$$

Here $\mathcal{J}_1(z)$ is a Bessel function of the first kind. To arrive at the equation in the main text, all that remains is to solve Eq. (S35) for $g(r, \omega)$. A particular solution to this equation that satisfies the required boundary conditions is the following:

$$g(z) = \frac{1}{2z}\left\{\mathrm{Shi}(z)\left((z^2+3)\cosh(z) - 3z\sinh(z)\right) - \mathrm{Chi}(z)\left((z^2+3)\sinh(z) - 3z\cosh(z)\right) - z\right\}, \tag{S39}$$

where $\mathrm{Shi}(z)$ and $\mathrm{Chi}(z)$ are the hyperbolic sine and cosine integral functions respectively. Comparing this expression for $g(z)$ with the approximate form suggested in Ref. [2] of $g(z) = c/(c + z^2)$ for some constant c – as shown in Fig. S1– we find that this corrected $g(z)$ is still well approximated by $g(z) \sim 7/(7 + z^2) = (14D/r^2)/(14D/r^2 - \mathrm{i}\omega)$.

We now turn our attention to the situation above T_c, and to the free vortex current at equilibrium. Following [3] we use Eq. (S24) and define the free vortex charge density $\rho_{\text{free}} = \sqrt{2\pi J_0}(n_+ - n_-)$. One obtains the following Fokker-Planck equation for the free charge density:

$$\frac{\partial\rho_{\text{free}}}{\partial t} = -\frac{2\pi J_0 D}{k_B T}\nabla_r\cdot[(n_+ + n_-)\mathbf{E}] + D\nabla^2\rho_{\text{free}}. \tag{S40}$$

Approximating $n_+ + n_-$ with a constant total vortex density n_f, and defining $\sigma = 2\pi J_0 D n_f/k_B T$, one can identify the following conserved current density:

$$\mathbf{j}_{\text{free}} = \sigma\mathbf{E} - D\nabla\rho_{\text{free}}. \tag{S41}$$

Moving to Fourier space, and using $\partial_t\rho_{\text{free}} = -\nabla\cdot\mathbf{j}$ to eliminate the charge density, one then obtains the expression for the current density used in the main text above T_c, in terms of longitudinal and transverse electric fields:

$$\mathbf{j}_{\text{free}} = \sigma\frac{\mathrm{i}\omega}{\mathrm{i}\omega - Dk^2}\mathbf{E}_L + \sigma\mathbf{E}_T. \tag{S42}$$

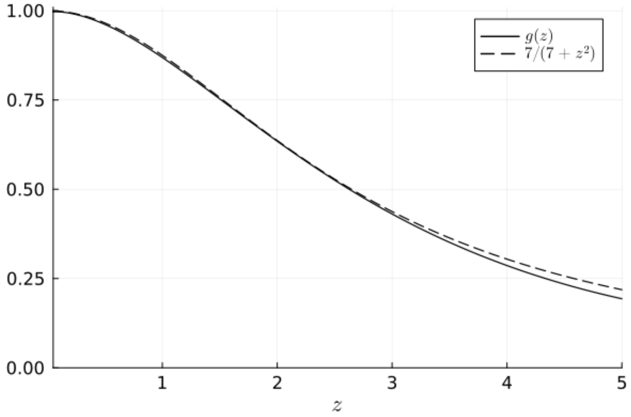

FIG. S1. Comparison of the exact solution of Eq. (S35 for the boundary conditions $g(0) = 1$, $\lim_{z\to\infty} g(z) = 0$ with the function $7/(7+z^2)$ used to approximate $g(z)$ in Ref. [2, 3]. Whilst the differential equation for $g(z)$ we derive conflicts with that presented in Ref. [2], the approximation for $g(z)$ adopted in that paper remains suitable despite the apparent error in that analysis.

In addition to the presence of a free current, and it's effect on the linear response of the system, the expression for the dynamical dielectric constant must also be modified. Above T_c, the spacing between unbound vortices is of the order of the correlation length ξ_+, and it is only below this length scale that it is sensible to discuss bound pairs. Thus the integration limits on the dynamical dielectric constant must be altered to read [3]:

$$\epsilon_b(\omega, k) = 1 + \int_{a_0}^{\xi_+} \mathrm{d}r \frac{\mathrm{d}\epsilon(r)}{\mathrm{d}r} e^{-k^2 r^2/32} g(r, \omega). \tag{S43}$$

As $\xi_+ \to a_0$, $\epsilon_b(\omega, k)$ becomes approximately independent of ω and k, and saturates at ϵ_c, the critical bulk value.

III. LINEAR RESPONSE OF SPIN WAVES AND VORTICES

To calculate the spin-density correlation functions presented in the main text, we follow Ref. [1] and Ref. [9] and obtain first the relevant linear response functions, and relate them back to the desired correlation functions using the fluctuation-dissipation theorem.

The most convenient setting for calculating these response functions is in the electromagnetic picture, where derivatives of the order parameter ϕ become electric and magnetic fields:

$$\mathbf{E}_L = \sqrt{2\pi J_0}\ \nabla\psi \times \hat{\mathbf{e}}_z, \tag{S44}$$

$$\mathbf{E}_T = \sqrt{2\pi J_0}\ \nabla\theta \times \hat{\mathbf{e}}_z, \tag{S45}$$

$$\mathbf{B} = \sqrt{2\pi\alpha}\ \dot{\phi}\ \hat{\mathbf{e}}_z. \tag{S46}$$

The correlations of both the electric and magnetic fields can both be derived from those of the vector potential $\mathcal{A}$, which we calculate in the Weyl (zero potential) gauge. As discussed in Ref. [9], correlations of the vector potential can be obtained from the imaginary part of the retarded Green's function for the vector potential $\mathcal{G}^R_{\mu,\nu}(\omega, \mathbf{r})$, which relates a current source to the macroscopic vector potential

$$A_\mu(\omega, \mathbf{r}) = -\frac{1}{\hbar c} \int \mathcal{G}^R_{\mu,\nu}(\omega, \mathbf{r} - \mathbf{r}') \bar{j}_\nu(\omega, \mathbf{r}') \mathrm{d}^2\mathbf{r}'. \tag{S47}$$

The current source $\bar{\mathbf{j}}$ should here be distinguished from the equilibrium current $\mathbf{j}_{\mathrm{eq}}$ due to the motion of free vortices; $\bar{\mathbf{j}}$ describes some fluctuation about equilibrium.

In Fourier-transformed variables, the Maxwell equations in a dielectric relating the vector potential to the total current read:

$$\left[k^2 - \frac{\omega^2}{c_0^2}\epsilon(\omega,k)\right] A_T(\omega,k) = \frac{2\pi}{c_0}\left(j_{\mathrm{eq};T} + \bar{j}_T\right), \tag{S48}$$

$$-\frac{\omega^2}{c_0^2}\epsilon(\omega,k) A_L(\omega,k) = \frac{2\pi}{c_0}\left(j_{\mathrm{eq};L} + \bar{j}_L\right). \tag{S49}$$

Below the critical temperature, the approximation is made to account for the effects of bound vortices through the dynamical dielectric function alone, and consider only spin-wave fluctuations [1]. This is achieved by taking the spin-wave field θ_0 that obeys the vortex-free Maxwell equations with a dynamical dielectric constant. θ_0 accounts for all the dynamics in ϕ below T_c, and is related to the emergent electric field by

$$\epsilon(\omega,k)\mathbf{E}_T = \sqrt{2\pi J_0}\nabla\theta_0 \times \hat{\mathbf{e}}_z. \tag{S50}$$

Thus in the BKT phase, in the absence of free vortices, the longitudinal component of the Green's function can be discarded, and with no equilibrium currents, one obtains:

$$\mathcal{G}_T^R(\omega,k) = \frac{2\pi\hbar}{(\omega^2/c_0^2)\epsilon(\omega,k) - k^2}. \tag{S51}$$

Note that factors of $\hbar$ here arise due to the use of the Kubo formula in arriving at Eq. (S47). We are interested in the classical limit, and will take $\hbar \to 0$ when appropriate. Using the fluctuation-dissipation theorem for bosonic particles, the fluctuations in the transverse vector potential are then:

$$\mathcal{C}_T(\omega,k) = -\coth\left(\frac{\hbar\omega}{2k_BT}\right)\mathrm{Im}\{\mathcal{G}_T^R(\omega,k)\}. \tag{S52}$$

Focusing on the regime $\hbar\omega \ll k_BT$, one returns to the classical limit. The transverse vector potential correlations can then be related to the correlation functions for the field ϕ (approximated by those of θ_0) and the out-of-plane spin density via:

$$\mathcal{C}_{\theta_0}(\omega,k) \approx \frac{\omega^2\epsilon_{\mathrm{Re}}(\omega,k)^2}{2\pi J_0 k^2 c_0^2}\mathcal{C}_T(\omega,k), \tag{S53}$$

$$\mathcal{C}_z(\omega,k) = \frac{J_0k^2}{2\pi c_0^2}\mathcal{C}_T(\omega,k), \tag{S54}$$

by making use of the Fourier transforms of the definitions (S44-S46). We have used the approximation that $\epsilon \approx \epsilon_{\mathrm{Re}}$ in Eq. (S53), as $\epsilon_{\mathrm{Im}} \ll 1$ is found by evaluation of the renormalization-group equations for a bare vortex chemical potential μ_0 larger than the spin-stiffness J. Explicit calculation of the vortex-antivortex creation energy shows that this is the case [6].

The in-plane spin-density correlations are given in terms of the ϕ correlations by:

$$\mathcal{C}_\perp(t,\mathbf{r}) = S^2 e^{\mathcal{C}_\phi(t,\mathbf{r}) - \mathcal{C}_\phi(0,\mathbf{0})}. \tag{S55}$$

To evaluate this, the approximation $\epsilon_{\mathrm{Im}} \approx 0$ is employed, and one finds below T_c:

$$\mathcal{C}_\phi(t,\mathbf{r}) \approx C_{\theta_0}(t,\mathbf{r}) \approx \frac{k_BT}{2\pi}\int_0^\infty \mathrm{d}k \frac{\epsilon_{\mathrm{Re}}(\omega,k)}{J_0k}\mathcal{J}_0(kr)\cos(\omega t) \tag{S56}$$

Here $\mathcal{J}_0(z)$ is a bessel function of the first kind.

Looking now above T_c, we can make use of the manipulations discussed in the previous section to include the effects of free vortices in the high spin-damping limit. We have

$$\mathbf{j}_{\mathrm{eq}}(\omega,k) = \sigma\frac{\mathrm{i}\omega}{\mathrm{i}\omega - Dk^2}\mathbf{E}_L + \sigma\mathbf{E}_T. \tag{S57}$$

Following the same manipulations as for below T_c, the transverse fluctuations are, in full:

$$\mathcal{C}_T(\omega, k) = \frac{4\pi k_B T c^2 \left(2\pi\sigma + \omega\epsilon_{\rm Im}(\omega, k)\right)}{\left[\omega^2\epsilon_{\rm Re}(\omega, k) - k^2 c_0^2\right]^2 + \left[2\pi\sigma\omega + \omega^2\epsilon_{\rm Im}(\omega, k)\right]^2}. \tag{S58}$$

The dynamical dielectric function includes only the effects of bound pairs, and is approximately constant ($\sim \epsilon_c$) and sufficiently far above T_c. The longitudinal correlations are:

$$\mathcal{C}_L(\omega, k) = \frac{\left(4\pi k_B T c^2/\omega^3\right)\left[2\pi\sigma\omega + \epsilon_{\rm Im}(\omega, k)(\omega^2 + (Dk^2)^2)\right]\left[(Dk^2)^2 + \omega^2\right]}{\left\{\epsilon_c[(Dk^2)^2 + \omega^2] + 2\pi\sigma Dk^2\right\}^2 + \left\{2\pi\sigma\omega + \epsilon_{\rm Im}(\omega, k)[\omega^2 + (Dk^2)^2]\right\}^2}. \tag{S59}$$

In practice, we make use of the approximation $\epsilon \sim \epsilon_c$ when evaluating the longitudinal correlations.

The mapping from the longitudinal vector potential correlations and the ψ field correlations is not straightforward. This is touched on in Ref. [1]: whilst $\nabla\psi$ is a well behaved function, ψ winds around each vortex, and is singular at each vortex core (allowing ψ to violate Stokes' theorem). The ψ field of a single vortex only has angular dependence, and must either contain branch cuts or be multi-valued. The Fourier-transform of this function is thus particularly ill-defined, and so the step $C_\psi = (\omega^2/2\pi J_0 k^2 c^2)C_L$ is not valid. However, as our system always contains a net zero vorticity, at large distances ψ must be a constant function, avoiding singularities in taking the Fourier transform at large scales. This does not solve the problem of branch-cuts or multiple values about each vortex however, and so we can only use C_L to approximate C_ψ up to a cut off momentum corresponding to length scales similar to the vortex spacing $\sim \xi_+$. This in turn implies a temperature scale above which our analysis is accurate for a given range of momenta, set by requiring $1/\xi_+ \gg 1/d$, which ensures no contribution to the relaxation rate from wave-numbers outside the valid range.

Above T_c, we have independent contributions from spin-waves and vortices (there are no off-diagonal components of the Green's function in the strong damping limit, and so vortices and spin-waves are decoupled). $C_{\theta_0}(t, \mathbf{r})$ and $C_\psi(t, \mathbf{r})$ are approximated by:

$$C_{\theta_0}(t, \mathbf{r}) \approx \frac{k_B T \epsilon_c}{2\pi J_0}\int_0^\infty {\rm d}k \frac{1}{k}\mathcal{J}_0(kr)e^{-\pi\sigma t/\epsilon_c}\left\{\cos\left[\frac{\Delta(k)t}{2}\right] - \frac{2\pi\sigma}{\epsilon_c\Delta(k)}\sin\left[\frac{\Delta(k)t}{2}\right]\right\}, \tag{S60}$$

$$C_\psi(t, \mathbf{r}) \approx \frac{k_B T}{2\pi J_0}\int_0^\infty {\rm d}k \frac{2\pi\sigma}{k(2\pi\sigma/\epsilon_c + Dk^2)}\mathcal{J}_0(kr)e^{-(2\pi\sigma/\epsilon_c + Dk^2)t}, \tag{S61}$$

$$\Delta(k) = \sqrt{4k^2 c_0^2/\epsilon_c - (2\pi\sigma/\epsilon_c)^2}. \tag{S62}$$

The removal of propagating spin-wave modes can be seen at the level of these correlation functions above T_c through the behavior of the function $\Delta(k)$. For $kc \gg \sigma$, this function is real, and so the integrand is oscillatory; in the limit $\sigma \to 0$ we recover the result for below T_c, albeit with a constant dielectric constant. For large σ however, $\Delta(k)$ is imaginary, and thus the integrand decays exponentially in time. We note also that in this limit of large σ (large vortex density), $C_{\theta_0}(t, \mathbf{r})$ and $C_\psi(t, \mathbf{r})$ are identical.

IV. NAÏVE FINITE SIZE SCALING IN LOW TEMPERATURE PHASE

In the low-temperature phase, as discussed in the main text, $\mathcal{S}(\omega)$ inherits algebraic low-frequency behavior from the correlations of the order parameter. In practice, these algebraic correlations will be sensitive to finite-size effects and other perturbations which introduce additional length scales into the system.

For a system of linear size L, the maximum coherence time is of the order L/c_0. The frequency dependence of $\mathcal{S}(\omega)$ at frequencies below the spin-wave maximum at $\omega \sim c_0/d$ is determined approximately by the integral:

$$\int {\rm d}t \cos(\omega t)(c_0 t)^{-\eta} e^{-c_0 t/L}, \tag{S63}$$

hence $\mathcal{S}(\omega \sim 0) \propto L^{1-\eta}$. Performing the integrals contributing to $\mathcal{S}(\omega)$ more precisely, the low frequency scaling presented in Fig. S2 is obtained, which is fitted to a power law with exponent $0.754 \approx 1 - \eta$ for the

chosen temperature.

[1] R. Cté and A. Griffin, Theory of the dynamic spin response function near the kosterlitz-thouless transition, Phys. Rev. B **34**, 6240 (1986).

[2] V. Ambegaokar and S. Teitel, Dynamics of vortex pairs in superfluid films, Phys. Rev. B **19**, 1667 (1979).

[3] V. Ambegaokar, B. I. Halperin, D. R. Nelson, and E. D. Siggia, Dynamics of superfluid films, Phys. Rev. B **21**, 1806 (1980).

[4] D. L. Huber, Dynamics of spin vortices in two-dimensional planar magnets, Phys. Rev. B **26**, 3758 (1982).

[5] J. B. Curtis, N. Maksimovic, N. R. Poniatowski, A. Yacoby, B. Halperin, P. Narang, and E. Demler, Probing the berezinskii-kosterlitz-thouless vortex unbinding transition in two-dimensional superconductors using local noise magnetometry, Phys. Rev. B **110**, 144518 (2024).

[6] J. M. Kosterlitz and D. J. Thouless, Ordering, metastability and phase transitions in two-dimensional systems, Journal of Physics C: Solid State Physics **6**, 1181 (1973).

[7] J. M. Kosterlitz, The critical properties of the two-dimensional xy model, Journal of Physics C: Solid State Physics **7**, 1046 (1974).

[8] A. P. Young, On the theory of the phase transition in the two-dimensional planar spin model, Journal of Physics C: Solid State Physics **11**, L453 (1978).

[9] E. M. Lifshitz and L. P. Pitaevskii, *Statistical Physics* (Pergamon, 1980) part 2, Sec. 75-77.

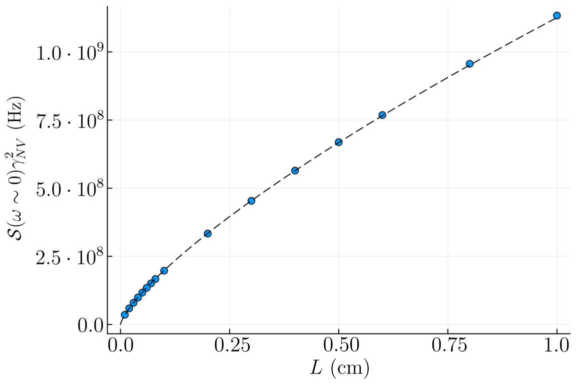

FIG. S2. Scaling of near zero value of $\mathcal{S}(\omega)$ calculated for increasing values of the linear system size L. Finite size effects are incorporated as a distance/time scale introducing exponential decay of correlations at late times and large distances. The resulting scaling with L is fitted to a power-law $\mathcal{S}(\omega \sim 0) = AL^B$, with $B = 0.754$, in close agreement with a rough calculation which predicts an exponent of $1 - \eta \approx 0.761$ at the chosen temperature of $T = 0.97T_c$.
